# Prediction of postoperative pain after radical prostatectomy

**DOI:** 10.1186/1472-6955-7-14

**Published:** 2008-12-09

**Authors:** Kerstin Wickström Ene, Gunnar Nordberg, Björn Sjöström, Ingrid Bergh

**Affiliations:** 1 The Sahlgrenska Acedemy at Göteborg University, Institute of Health and Care Sciences, Gothenburg, Sweden; 2 Departments of Anesthesiology and Intensive Care, Sahlgrenska University, Sweden; 3 University of Skövde, School of Life Sciences, Skövde, Sweden

## Abstract

**Background:**

There is a belief that the amount of pain perceived is merely directly proportional to the extent of injury. The intensity of postoperative pain is however influenced by multiple factors aside from the extent of trauma. The purpose of the study was to evaluate the relationship between preoperative factors that have been shown to predict postoperative pain and the self-reports of pain intensity in a population of 155 men undergoing radical prostatectomy (RP), and also to investigate if previous pain score could predict the subsequent pain score.

**Methods:**

The correlation between potential pain predictors and the postoperative pain experiences during three postoperative days was tested (Pitmans' test). By use of a logistic regression analysis the probability that a Visual Analogue Scale (VAS) score at one occasion would exceed 30 mm or 70 mm was studied, depending on previous VAS score, age, depression and pain treatment method.

**Results:**

Age was found to be a predictor of VAS > 30 mm, with younger patients at higher risk for pain, and preoperative depression predicted VAS > 70 mm. The probability that VAS would exceed 30 mm and 70 mm was predicted only by previous VAS value. Day two however, patients with epidural analgesia were at higher risk for experiencing pain than patients with intrathecal or systemic opioid analgesia.

**Conclusion:**

The results show that it would be meaningful to identify RP patients at high risk for severe postoperative pain; i.e. younger and/or depressive patients who might benefit from a more aggressive therapy instituted in the very early postoperative period.

## Background

Pain is one of the major concerns in the postoperative care, not only because of the suffering it causes, but also because of its potential association with the process of recovery [1]. There is a belief that the amount of pain perceived is merely directly proportional to the extent of injury [2]. The severity of postoperative pain is however influenced by multiple factors aside from the extent of trauma [3]. Despite of identical surgical procedures, there is postoperatively a large variation in the pain experience and analgesic requirement [4].

There can be several explanations for this and studies have shown that the threshold for pain is different in different patients [5].

In patients with prostate cancer, radical prostatectomy (RP) is the most common technique for removing the prostate gland and it is a procedure performed with increasing frequency [6]. In RP, an incision in the lower abdomen, from the pubic bone to the navel, is used to reach the prostate gland. The prostate gland is detached from the bladder; the overlying veins, seminal vesicles and vas deferens are also removed. The urethra is reconnected to the bladder and a catheter is inserted into the penis through the urethra into the bladder and is left in place until the reconnection heals. Drains will be put into the abdomen and will be left in place for a couple of days to excess fluids, such as blood and urine [7]. Postoperative pain after RP can be moderate to severe but is often of rather short duration [8].

Different factors have been found to influence the intensity of postoperative pain. In previous studies we have shown that the method for postoperative pain treatment after RP was of importance [9,10]. A pre-surgical intrathecal bolus dose of morphine and local anesthetics (ITA) was found to be superior to a continuous epidural analgesia (EDA) for pain relief, and it also shortened hospital stay [10].

Preoperative expectations of pain have been found to correlate with the postoperative pain experience [11,12]. Perceived control, e.g. the perception of, or belief in, the availability of a response that can reduce or limit pain, has been associated with less pain reports and an increased pain tolerance [1]. It has previously been shown that patients who are more internal, e.g. believing that they can influence and are responsible for their own health [13] have lower pain scores and use less postoperative morphine [14].

Psychological factors such as anxiety and/or depression have been considered as important predictors of postoperative pain [4,15]. Age has also been found as a predictor, with younger individuals being at higher risk for moderate to intense pain [15]. Identification of patients at high risk for severe postoperative pain and giving those patients special attention would be desirable. Patients with good analgesia are more co-operative, recover more rapidly and leave hospital sooner. They also have a lower risk for prolonged pain after surgery [16].

Nurses are in a unique position to supervise and assist patients in pain and in the treatment thereof, considering the extensive time nurses spend with the patients when compared with other health-team members [17]. Nursing pain management involves a number of activities; assessing pain and deciding whether to administer analgesics, selecting one of different analgesics and choosing the route of administration. Nurses are also responsible for monitoring the effect of medication which is prescribed and administered in a variety of ways, including PRN (pro re nata, as needed/requested), EDA and ITA [18].

Several studies have investigated the relationship between pre-surgical clinical factors and postoperative pain. The influence of previous pain score (Visual Analogue Scale, VAS-value) on the next-coming pain scores has not earlier been studied in this group of patients i.e. if patients who are in pain directly after surgery continue to be in pain during the postoperative recovery. The purpose of the present study was therefore to evaluate the relationship between preoperative factors that have been shown to predict postoperative pain and the self-reports of pain intensity in a population of 155 men undergoing RP, and also to investigate whether a previous pain score could predict the subsequent pain score.

## Methods

### Design/Sample

The study was a prospective, explorative study conducted from January 2003 to June 2004. After approval was obtained from the Ethics Committee of the Sahlgrenska University Hospital, Sweden, patients on the waiting-list for RP, were recruited for the study.

### Instruments

#### Demographic form

The demographic form comprised questions about age, civil status, education, employment, time on waiting list previous surgical experiences, previous postoperative pain experiences and postoperative pain expectations.

Data of physical status classification according to the American Society of Anaesthesiologists (ASA score), psa, weight, surgery time, intra-operative bleeding, pain treatment method and opioids consumption were collected from the patients' records.

#### Visual Analogue Scale (VAS)

Pain was measured with a VAS (0–100 mm) on which the patients' pain intensity was represented by a point between the extremes of "no pain at all" and "worst pain imaginable". The simplicity, reliability and validity of the instrument make the VAS a good tool for describing pain severity or intensity [19].

The analysis of the VAS scores is a frequently discussed matter. In studies using the VAS, a score of more than 30/100 mm is often used as a limit to indicate inadequate analgesia and a VAS score of more than 70/100 mm is a common breakpoint for defining severe pain [20]. A mean pain score of VAS > 30 mm has been found to have a significant effect on general activity and mood and VAS ≤ 30 mm thus should be maintained to optimize the patients' functional status [21].

#### Hospital Anxiety and Depression Scale (HAD)

The HAD scale [22] has been found to be a reliable (Cronbach's alpha > 0.80) instrument for assessing the symptom severity of anxiety disorders and depression in somatic, psychiatric and primary care patients as well as in a general population [23]. The instrument is a 14-item, self-administered rating scale that produces two sub-scales, one measuring anxiety (HAD-A) and the other measuring depression (HAD-D). Each item has four response categories, reflecting a continuum of increasing level of emotional distress. Thus, HAD ≤7 indicates no anxiety (HAD-A) or depression (HAD-D), HAD 8 – 10 indicates possible anxiety or depression, and HAD ≥ 11 indicates probable anxiety or depression.

#### Multidimensional Health Locus of Control (MHLC)

MHLC measures expectancies about control, and was developed for prediction of health related behaviour [13]. The scale is an 18-item questionnaire measuring the subjects' beliefs concerning three dimensions of control of health outcomes; i.e. "internal" (IHLC), "powerful others" (PHLC) and "chance" CHLC). All of the dimensions are independent of one another and there is no total MHLC score. People who believe they can influence and take responsibility for their own health are labelled as "internals". Those who score high on the "powerful others" subscale are likely to rely on others (e.g. doctors and nurses) to control their health. Finally, those who score high on the "chance" subscale are not likely to rely on their own actions or the action of others because they believe that their health rather is a matter of chance. There are six statements for each dimension. Each statement is rated on a scale from 1–6 with 1 indicating "strongly disagree" and 6 indicating "strongly agree", making the range of scores 6–36 for each dimension. The scale is reliable with a Cronbach alpha in the 0.60–0.75 range [24].

### Procedure

Three weeks before surgery, consecutive patients on the waiting-list for RP received a letter with written information about the study. Patients willing to participate signed and returned a consent form. At the same time the patients answered the form about demographics and the MHLC questionnaire. The day before surgery, the patients answered HAD and they were informed about the VAS. Patients were asked, by the author (KWE), about pain at four hours after surgery and "worst pain" experienced during the last 24 hours at intervals of 24, 48 and 72 hours. The patients were asked to put a mark on a 100 mm line, representing "worst pain" experienced.

### Pain treatment routines

Initially EDA was the routine treatment for postoperative pain in these RP patients. About a year after the beginning of the study, and after evaluating EDA as an ineffective method for pain treatment in this group of patients, the method for postoperative analgesia was shifted to ITA. Study patients who were deemed unsuitable for either EDA or ITA, received systemic opioid analgesia (SOA) for pain relief.

After the operation, the patients were supposed to stay in the postoperative anaesthesia care unit (PACU) until the pain relief was sufficient. When the patients returned to the ward, each EDA and ITA patient had a checklist for basic and specific controls e.g. VAS, sedation, motor function and hemodynamics. The patients given only SOA for pain relief did not have any special protocol for pain assessment. The recommended pain level was to be VAS < 30–40 mm on a 100 mm scale. All patients received paracetamol (1 g × 3–4) starting preoperatively and continuing postoperatively until the patients left the hospital. If pain relief was not sufficient on the ward, additional doses of ketobemidone (equianalgesic morphine type of opioid analgesia) were given systemically on a PRN (as requested) basis. Oral rescue analgesics (tramadol and NSAID's) were given at the discretion of the surgeon on the ward.

### Statistical analysis

SPSS (version 14.0) for data analyses was used to analyze the data. Continuous variables are presented as means and standard deviation, and categorical data are presented as number and percent. By use of a non-parametric test (Pitmans' test) [25] the correlation between "worst pain" and different possible predictors have been tested. Pitman's test is a non-parametric test not based on ranks but on the original values. By use of logistic regression analysis we estimated the probability that VAS at one occasion would exceed 30 mm or 70 mm and the result is presented by graphs calculated from the beta coefficients. The cut-off 30 mm was chosen because that is a limit for treatment. All tests were conducted at the 5% significance level.

## Results

### Demographics

Of the 181 patients who were invited, 155 patients (86%) gave informed consent to participate. The mean age of the patients was 63 years (range 43–73). Most of the subjects (89%) were married, and about one third had elementary education. Half of the patients were retired. After being diagnosed with prostate cancer, 41% of the patients had to wait more than three months for their operation. Data from the patients' records are presented in Table 1.

**Table 1:** Data collected from the patients' records

ASA-class	
I	53 (34)
II	93 (60)
III	9 (6)
PSA	9.5 ± 7 (1.5–60)
Weight	84 ± 11 (55–122)
Duration of surgery (min)	140 ± 31 (65–223)
Intraoperative bleeding (ml)	1670 ± 1000 (200–5800)
Pain treatment	
EDA	90 (58)
ITA	50 (32)
SOA	15 (10)
Opioid consumption (mg)	
EDA	9.8 ± 12 (0–67.5)
ITA	7.3 ± 11 (0–63)
SOA	26.8 ± 23 (0–75)

Continuous data are presented as mean ± SD and range and categorical data as n (%).

EDA = epidural analgesia, ITA = intrathecal analgesia, SOA = systemic opioid analgesia

A pain level higher than VAS 30 mm during the three postoperative days was predicted by age (p = 0.044) and there was a correlation (p = 0.016) between age and "worst pain", with younger patients reporting higher pain scores than older (Table 2). There were no correlation between age and the opioid consumption. We found no correlation between any of the other demographic variables and the experienced level of postoperative pain.

**Table 2:** Univariate analysis of the association between potential pain predictors and postoperative pain

Variable	Two-sided p-value
Psa	> 0.30
ASA	> 0.30
Weight	> 0.30
Age	0.016*
Marital status	0.30
Employment	0.13
Education	0.20
Time on waiting-list	> 0.30
Previous surgery	> 0.30
Previous pain experience	> 0.30
Pain expectation	0.29
Surgery time	> 0.30
Intra-operative bleeding	0.19
HAD anxiety	0.073
HAD depression	0.020*
MHLC internal	> 0.30
MHLC chance	> 0.30
MHLC powerful others	0.14

*p < 0.05

### Pain expectations and pain experiences

One hundred and five (68%) patients had previously undergone surgical procedures. Of these patients, 36 (44%) had experienced moderate/severe pain. Moderate/severe pain after the RP was expected by 121 (78%) patients and was experienced by 105 (68%) patients. Patients with previous experience of postoperative pain expected higher pain scores (p < 0.01), though this was not actually experienced. Only four patients reported some kind of pain (e.g. back problems) before surgery.

### Perceived control, anxiety and depression

The HAD questionnaire was answered by 133 (86%) patients. Possible or probable preoperative anxiety was reported by 33 (25%) of these patients (Table 3). There was no significant correlation between preoperative anxiety (HAD-A) and postoperative pain (p = 0.073). The incidence of preoperative depression was lower (n = 16, 12%), but depression was found to correlate with postoperative pain (p = 0.020) and depression predicted a pain level higher than VAS 70 mm (p = 0.0071). We found no correlation between age and anxiety/depression. No significant correlations were found between the different dimensions of MHLC and the postoperative pain experience.

**Table 3:** MHLC and HAD scales

MHLC (n = 145)	
IHLC	18 ± 7
PHLC	11 ± 12
CLOC	20 ± 7
HAD-A (n = 133)	5.2 ± 4
HAD-D (n = 133)	3.2 ± 3
HAD-class A	
no anxiety	100 (77)
possible/probable anxiety	33 (25)
HAD-class D	
no depression	117 (88)
possible/probable depression	16 (12)

Multidimensional Health Locus of Control (MHLC), with the dimensions; internal (IHLC), powerful others (PHLC) and chance (CHLC), scores range from 6–36. Hospital Anxiety Scale (HAD-A = anxiety, HAD-D = depression). Continuous data are presented as mean ± SD and categorical data as n (%).

### Pain experience and pain treatment methods

**Figure 1:**
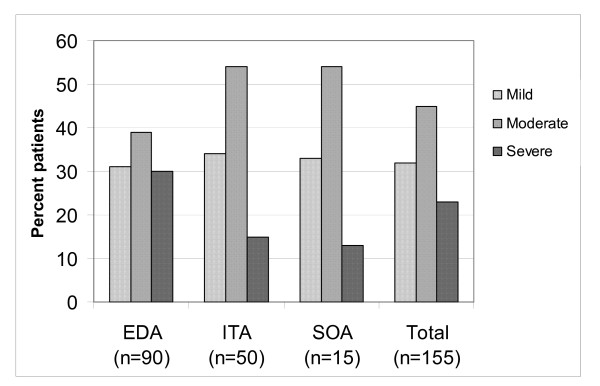
Differences among pain treatment methods with regard to "worst pain" scores.

With regard to the first three postoperative days, 50 patients (32%) reported mild, 69 (45%) moderate and 36 (23%) severe pain for one or more days (Figure 1). Mean pain score, independent of pain treatment method, was after four hours 11 ± 20 (range 0–100), day one 40 ± 26 (range 0–100), day two 32 ± 28 (range 0–100) and day three 20 ± 23 (range 0–90).

Mean PACU-time was 14 ± 7 hours. Patients with severe pain had the longest PACU-time (18 h vs 15 h for moderate pain and 12 h for mild pain, p < 0.01). Twenty (13%) patients had a pain score > VAS 30 mm after four hours. During the rest of day one, pain subsequently increased with 85 (55%) patients reporting a pain score > VAS 30 mm. In the twenty patients with VAS > 30 mm after four hours, pain increased in 14 (70%) patients.

Of the 155 patients, 90 (58%) received EDA, 50 (32%) ITA, and 15 (10%) received SOA for their postoperative pain relief (Figure 1). There were more patients in the EDA group than in the other two groups, who reported high pain scores (p < 0.05). Days one and three, the pain treatment methods had no influence on the pain experience. Day two however, the EDA patients reported higher pain levels (p < 0.001) than the patients with ITA or SOA (mean VAS score 40 mm vs 24 mm and 18 mm). Patients who only received SOA for pain relief used more opiods (p < 0.001) than the EDA and ITA patients (mean 27 mg vs 10 mg for the EDA- and 7 mg for the ITA patients).

### How well do previous VAS values predict the next coming value?

The correlation coefficients (r) between VAS at four hours and the rest of day one were 0.52 (p < 0.001), Pitman's test [25], between VAS day one and day two 0.47 (p < 0.001) and between VAS day two and day three 0.55 (p < 0.001).

**Figure 2:**
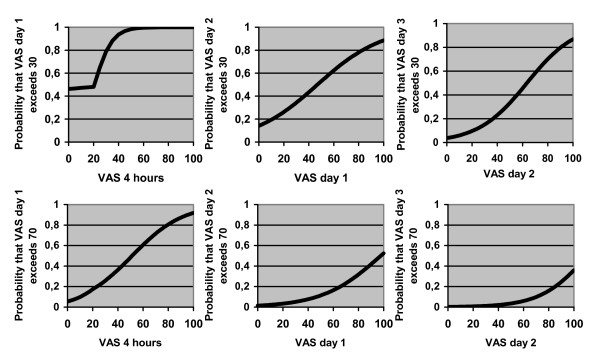
**The figures show the probability that VAS exceeds 30 mm and 70 mm days one, two and three depending on previous VAS values.** If e.g. the VAS value is 40 after four hours, the probability that VAS exceeds 30 mm day one is 95% and the probability that VAS exceeds 70 mm is 40%.

**Figure 3:**
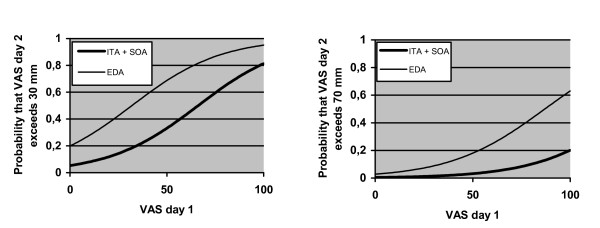
The figures show that patients with EDA were at higher risk for experiencing pain levels > 30 mm or > 70 mm day two after surgery compared to patients with ITA and SOA.

By use of logistic regression analysis the probability that VAS at one occasion would exceed 30 mm or 70 mm was studied, depending on previous VAS values, age, depression and pain treatment methods. The probability that VAS day one would exceed 30 mm (p = 0.017) and 70 mm (p = 0.0103) was predicted only by VAS after four hours (Figure 2). The prediction could not be improved by including age and depression. The probability that VAS day two would exceed 30 mm was predicted by VAS day one (p < 0.0001) and EDA (p < 0.001) and that VAS day two would exceed 70 mm was predicted by VAS day one (p < 0.001) and EDA (p = 0.016) (Figure 3). The prediction could not be improved by including VAS at four hours, age, depression, ITA or systemic opioids. The probability that VAS day three would exceed 30 mm (p = 0.0001) and 70 mm (p = 0.0051) was predicted only by VAS day two. The prediction could not be improved by including VAS at four hours, VAS day one, age, depression or pain treatment method.

## Discussion

The study has demonstrated that in RP patients, age and depression are found to be predictors of postoperative pain. A pain level > VAS 30 mm at one occasion during the three postoperative days was predicted by age, and a pain level > VAS 70 mm was predicted by depression. From a treatment perspective we wanted to predict whether the patient needs treatment in the next future, so that we, in the best case, can give the treatment before the pain has increased above 30 mm on the VAS scale. For that purpose we did not need to make a prediction at baseline or by use of the baseline values. We needed to make a prediction with a time horizon of a few hours only to get the opportunity to treat the patient. By use of logistic regression analysis we found that the only factor that could predict pain was the previous VAS score, except for day two, when we found that patients with EDA reported significantly higher pain scores than the patients with other pain treatments. A surprisingly finding was that as much as one out of four patients (23%) of the total sample and 30% of the EDA patients experienced severe pain.

Patients undergoing the same type of surgery are often given the same type of postoperative pain treatment, but age-related pharmacokinetic and pharmacodynamic factors may influence the variance in analgesic needs [26]. With a univariate analysis, age was found to be predictor of pain > 30 mm, with younger patients at a higher risk of experiencing pain. Others have also found younger patients to report higher pain scores than older ones [12,15,27], possibly reflecting that young patients with a cancer diagnosis may experience greater distress than older patients because of the effect of serious illness on their life and therefore report higher pain levels [4]. In patients using postoperative patient-controlled analgesia, age is found to be the best predictor of postoperative morphine requirements [26]. We found no correlation between age and the opioid consumption in the present study.

Preoperative emotional variables such as anxiety and depression have been found to influence pain experience. In this study we found depression to be of importance for a pain level > VAS 70 mm. In a previous study, we found depression to be a predictor of pain in RP patients, and that preoperative depression also affected pain and depression after discharge from hospital [28]. Even in multivariate studies, depression has been shown to be a strong predictor of postoperative pain [15,29]. Pre-operative state anxiety has repeatedly been shown to correlate with post-operative pain severity [15,27]. In this study we only found a tendency that preoperative anxiety would influence the postoperative pain. High expected pain severity has been found to predict severe pain [11,12]. This was not confirmed in the present study when more patients expected moderate/severe pain to a higher degree than was actually experienced.

Regarding MHLC, it has previously been shown that patients who are more internal, i.e. who believe that they can influence and are responsible for their own health [13], have lower pain scores and use less postoperative morphine [14]. This was however not confirmed in the present study, where we found no significant correlation between any of the different dimensions of the MHLC instrument and the pain intensity. The low predictive power of the MHLC variables might result from a relatively low sensitivity of the general MHLC scales to various problems of post-surgery patients [30].

In a multivariate analysis model we found the only predictor of the severity of postoperative pain to be the previous VAS value. Seventy percent of the patients with a pain score > VAS 30 mm at four hours after surgery continued to be in pain. Pre-emtive analgesia is a frequently discussed matter but studies comparing pre-incisional with post-incisional treatment have failed to provide convincing evidence for the value of preemptive analgesia [31]. Kissin [31] discusses the definition of preemptive analgesia and defines it as "treatment that prevent establishment of central sensitization caused by incisional and inflammatory injuries; it starts before incision and covers both the period of surgery and the initial postoperative period". This means that effective blockade of noxious stimuli during the initial postoperative period reduces subsequent postoperative pain [32]. Patients who wake up after surgery with insufficient pain relief should be treated immediately to avoid further pain.

Several studies have reported EDA with local anaesthetics combined with opioids as a safe and effective method [33,34]. Some studies though, report a fair amount of different complications to the epidural treatment, not only with hypotension, parestesis and motor blockade, but of technical complications and premature removal [35,36] which also may result in insufficient pain relief. In the present study, patients with EDA reported higher pain score day two than patients with ITA or systemic opioids. We have previously reported on EDA as an insufficient method for pain management in RP patients with the average patient not experiencing a pain score that was sufficient until day three [9]. The findings in our previous study were related to problems/barriers associated with the individual epidural pain treatment regime and an inadequate service response in general to patients with moderate or severe pain. Others have reported insufficient pain relief in patients with postoperative EDA, with one third of the patients suffering from significant pain [37]. In contrast to these findings, Caumo et al [15] found EDA to protect against moderate to intense postoperative pain. The method for treatment of postoperative pain at our hospital is today changed to ITA [10].

Nurses play an important role in the pain management. They assess and document pain, decide whether to administer analgesics, and they monitor the effect of medication which is prescribed and administered in a variety of ways. In the present study supplemental systemic opioids were supposed to be given on a PRN basis until pain relief was sufficient. There is evidence that nurses are conservative when making decisions about opioid dosing and frequency of administration [38]. Consistent with our findings, it has previously been reported that patients with mild pain receive significantly lower doses of opioids and even if higher doses of opioids are given to those with severe pain, these doses are not titrated to optimal reduction of pain severity [39]. Nurses do not always titrate opioids appropriately and do not increase subsequent doses of opioids when the previous dose has been safe but ineffective [40]. Under-medication of pain is often the result of the nurses' failure to involve patients in pain decisions and also of a lack of trust regarding the patients' reports concerning the quality of their pain [41]. Nurses have to be aware of the fact that in general, younger patients need more opioids than older patients. For their perception of patients' pain, doctors generally rely on nurses to report pain in their patients. If nurses underestimate and/or do not document pain, this is likely to result in under-medication in many patients [42].

## Limitations

The method for postoperative pain relief was gradually shifted from EDA to ITA which may have affected the study result. However, the patients were not assigned to different pain treatment methods depending on patient factors. All of the patients were men, with the same diagnosis, who underwent the same kind of surgery and all patients were interviewed about their pain experiences in a consistent way and the result should therefore be valid.

Patients were asked once a day about their "worst pain" experienced during the preceding 24 hours, though it was not possible to meet all patients for an interview more often than once a day. This method of pain assessment with an overall daily retrospective estimation may overlook periods with more or less pain [43]. However, others have reported about a significant correlation between moderate/severe pain when frequently reported and when measured once daily [44].

## Conclusion

The results of the present study show that it would be meaningful to identify at least a subgroup of RP patients at high risk for severe postoperative pain. This could be related to an insufficient treatment *per se*. It could however also imply that pain once manifested is not easily converted despite a generous analgesic treatment. This may be more evident in subgroups of patients, e.g. younger and depressive patients, who might benefit from a more aggressive therapy instituted in the very early postoperative period. When using EDA, the patients and the epidural catheters should be observed closely and systematically, by adequately trained members of the staff competent to detect inadequate analgesia and potential problems with the use of EDA, and to implement proper solutions [35]. This requires proper training of staff outside the formal anaesthetic unit, but still this will not exclude the continuous need of an adequate communication between the anaesthesiologists, who insert the epidural catheter, and the ward staff who cares for the patients postoperatively.

## Competing interests

The authors declare that they have no competing interests.

## Authors' contributions

KWE and BS were responsible for the study design. KWE performed the data collection. All authors contributed to the data interpretation, helped to write the manuscript and approved the final version.

## Pre-publication history

The pre-publication history for this paper can be accessed here:


